# Transcultural nursing: a qualitative analysis of nursing students’ experiences in a multicultural context in North-Eastern Namibia

**DOI:** 10.1186/s12912-024-01773-8

**Published:** 2024-02-15

**Authors:** Vistolina Nuuyoma, Sitembile Muvumwaeni, Leonard Chihururu

**Affiliations:** School of Nursing and Public Health, University of Namibia, Rundu Campus, Rundu, Namibia

**Keywords:** Cultural competence, Cultural diversity, Clinical practice, Multiculturalism, Nursing students, Transcultural nursing

## Abstract

**Background:**

Culture is a fundamental concept in healthcare settings due to the reason that care provided to patients is holistic and encompasses their perspectives on health, which are greatly influenced by the patients’ cultures. To prepare culturally competent nursing graduates, it is important to understand the experiences of students on transcultural nursing during clinical practice. However, there are limited studies that have explored experiences of students on transcultural nursing, specifically those located in multicultural societies. In addition, studies focus on experiences of international students who visit with student - exchange programme. Nevertheless, their experiences of transcultural nursing may differ since they are not residents and have limited exposure to transcultural nursing, and therefore cannot be generalized to undergraduate resident students. This study aimed to explore and describe transcultural nursing experiences of nursing students during clinical practice at an intermediate hospital in north-eastern Namibia.

**Methods:**

Descriptive and explorative qualitative designs were used, data were collected via individual interviews from 16 final year nursing students, who were sampled using maximum variation purposive and snowballing sampling. During data collection, an interview guide was used together with audiotape and field notes. Data analysis followed Tesch’s eight steps in qualitative coding process. Trustworthiness was ensured using four principles of Lincoln and Guba, Moreover, ethical clearance and permission were granted by research ethics committees from two institutions.

**Findings:**

Four main themes emerged as findings of the study are: nursing students’ exposure to different cultural practices and beliefs; personal feelings experienced by nursing students during transcultural nursing; challenges experienced by students during transcultural nursing; and nursing students coping mechanisms.

**Conclusions:**

Nursing students had mixed experiences on transcultural nursing which touch on aspects such as exposure to cultural aspects, personal feelings, challenges, and coping mechanisms. These findings are useful in helping nurse educators, clinical mentors, students, and future researchers to understand experiences of students on transcultural nursing. Consequently, assist in enriching transcultural nursing issues in curricula and for adequate preparation of graduates to become culturally competent when providing nursing care.

**Supplementary Information:**

The online version contains supplementary material available at 10.1186/s12912-024-01773-8.

## Introduction

Culture refers to dynamic system that creates, and is created by people, places, and practices [1]. It consists of sets of values and beliefs, or a cluster of learned behaviors that are shared with others in a particular society therefore, giving people a sense of belongingness and identity [2]. Culture is a fundamental concept in healthcare settings due to the reason that care provided to patients is holistic and encompasses their perspectives on health, which are greatly influenced by the patients’ cultures [3]. The P- Model designed by Causadias [1] to define culture consists of four concepts, which are people, places, practices, and power. People in culture refers to population dynamics, social relations, and culture in groups. Places indicate institutional influences, ecological dynamics, and culture in contexts. Practices pertain to community engagement, participatory dynamics, and culture in action. While power refers to forcing others into compliance, controlling access to space, and behaving as desired. Most definitions of culture found in literature pointed out on values and norms shared among members of a social group as part of culture and that they create expectations for certain behaviors [4]. Organizations such as health care facilities are generally classified as multi-cultural entities due to various professional and specialized teams that operate within them, therefore, creating subcultures within them. A subculture is typically comprised of people who have a distinct identity and yet are related to a larger cultural group. Individuals in subcultures share same ethnic origin and physical characteristics with the larger cultural group however, they conform to norms, values and practices affiliated to their occupational and societal groups [3].

Due to globalization of healthcare, nurses and midwives throughout the world are increasingly expected to come into contact with patients, families and colleagues from diverse cultures and backgrounds [5], consequently, providing transcultural nursing. The concept transcultural nursing was coined by Madeline Leininger in the 1950s, hence the development of Theory of Transcultural Nursing. The theory emphasized that patient care and communication exhibited by nurses should actively incorporates the patient’s background, values and beliefs into every step of the nursing process [6]. It also involves associating differences and similarities between cultures and relates them to caring values and life practices in order to envisage individual’s care needs and promote culturally congruent care. It focuses on the universality of human caring and analysis of diversity as well as dynamics of world in relation to human beliefs, behaviors and their caring values [7]. Leininger appealed for nurses to develop knowledge on and to possess ability for provision of culturally competent care [8]. Although the ultimate aim of nursing should be to provide a caring service that respects people’s cultural values and lifestyles, it is also influenced by nurses’ own values, beliefs and customs since they have own cultures that are different from patients [9]. Therefore, the nurse’s self-awareness can be the starting point to understand the patient’s culturally and master the skill of providing culturally competent care.

Through clinical nursing practice, nursing students become part of the healthcare and therefore expected to be culturally competent when providing transcultural nursing care. According to Çingöl et al., [10], receiving culturally appropriate healthcare service is a human right, and every individual has the right to receive nursing care in accordance with their culture. It is therefore expected that health professions education prepares well equipped graduates who adopt a neutral and positive approach to provide quality care tailored to cultural contexts of different people.

A review by Qui and Jiang [11] revealed that nursing students benefits from transcultural nursing by learning global nursing knowledge and experience cross-cultural interpersonal relationships. The same review also revealed that nursing students are prone to psychological problems and have encountered language problems during transcultural nursing. Other challenges of provision of transcultural care experienced by nursing students are intrapersonal struggle, cultural conflicts, varied expressions of discomforts such as pain and suffering, as well as personal and organizational constraints [12]. In a study by Okeya [13], findings revealed that nursing students lacked confidence, sufficient knowledge and experiences required to provide quality care for ethnic minority patients who are from diverse cultures.

Namibia is a multicultural society with 13 ethnic groups that practice diverse cultures. Of the 13 groups, there is a Kavango ethnic group which predominantly occupies Kavango east and west regions, which is further divided into five sub-ethnic groups namely: Mbukushu, Sambyu, Rukwangali, Mbunza and Gciriku. Rundu town in Kavango east region has recently become an ethnically diverse town with its population having increased drastically. Due to this population increase and migration of people from diverse ethnic groups in Namibia and other countries, the health care facilities have become multicultural settings and have incorporated transcultural nursing in provision of healthcare services. The observation at an intermediate hospital in Rundu is that there are language barriers between nurses and patients due to multiculturalism and lack of common language spoken by all residents.

Many nursing schools include intercultural nursing courses in their curricula and also providing short training for nurses to be culturally sensitive, capable of providing culturally adequate care, and who have improved empathy skills [10]. Course objectives incorporated in curriculum to prepare students for transcultural nursing are cultural awareness, cultural knowledge, cultural skills and cultural encounters [7]. These objectives are derived from components proposed by Campinha-Bacote Model to be included in the curriculum in order to develop culturally sensitive graduates [14]. In Namibia, nursing curricula incorporate transcultural nursing aspects in courses like nursing ethics and professional practice, general nursing science, foundations of sociology and community health nursing science. While Prosen [7] suggested learning and teaching activities such as clinical practice, cultural immersion experience, reflective writing and mentoring to be included in curriculum to promote cultural encounters, which is one of the vital aspects in transcultural nursing. It is however a concern that most courses in Namibia merely touch on the importance of recognizing and understanding diverse cultures without experiential learning outcomes related to cultural encounters. Moreover, it is not known how undergraduate nursing students in north-eastern Namibia experience transcultural nursing during clinical practice. There seems to be limited studies that have explored experiences of students on transcultural nursing, specifically those located in multicultural societies. The only study conducted in Namibia on experiences of transcultural nursing focuses on international nursing students who visit through the student - exchange programme [15]. Their experiences may differ due to the reason that they are not residents and have limited exposures to clinical practice, and therefore cannot be generalized to undergraduate nursing students who are residence of the country.

### Aim of the study

The aim of this study was to explore and describe transcultural nursing experiences of nursing students during clinical practice in a multicultural context in north-eastern Namibia.

## Methods

### Study design

This study followed a qualitative descriptive– explorative approach as its research design. This design was chosen to allow nursing students to share their experiences of transcultural nursing and how these experiences impact on their clinical practice at an intermediate hospital in a multicultural context. This design follows the assumptions of a social constructivist philosophical research paradigm. Moreover, the Consolidated Criteria for Reporting Qualitative Research (COREQ) was followed as a reporting guideline [16].

### Study setting

This study was conducted at an intermediate hospital, a 530-bed capacity health care facility situated in Rundu town, Kavango east region, Namibia. The hospital is 716 km from Windhoek, which is a capital city of Namibia. The facility serves as a referral point for five district hospitals located in three regions in eastern Namibia. It includes departments such as outpatient and casualty; high care and isolation care ward; surgical ward; medical wards separated for females and males; maternity and neonatal care ward; paediatric ward; tuberculosis ward; operating theatre and medical sterilizing unit. To emphasize experiential learning, which is part of constructivism educational philosophy as followed by many nursing schools [17], nursing students are placed for clinical practice at those departments, for a duration of two– one month, depending on the academic calendar.

Kavango east region border with Angola, separated mostly by Kavango river, while on the northeast, there is Caprivi strip, which share border with Botswana, Zambia and Zimbabwe. Patients and clients who get health care services from an intermediate hospital where students are placed for clinical practice are from various cultures in Namibia and neighboring countries such as Botswana, Angola, Zambia, and Zimbabwe since the hospital is located at the border town, which is a transit point for many non-Namibians. Kavango east region also receives other African and international visitors due to tourist attractions such as river, game parks, museums, unique vegetations and cultures. In addition, people who originates from China, India and Pakistan become inhabitants of Kavango east region, mainly for business and trading purposes. Nursing students are placed to be part of the team rendering care to these patients and clients.

Conversely, most nursing schools in Namibia demonstrates intercultural relations through catering for students from different cultural backgrounds as well as international students. This has led to the nursing school at a public university being multicultural accommodating students from the Namibian tribes of Okavango, Caprivi, Owambo, Ovahimba, Ovaherero, Nama and Damara, tribes originate from countries like Zimbabwe, Zambia, and Angola.

### Study population and sample

The target population was final year nursing students in the Bachelor of Nursing Science (clinical) honours programme at the public university campus located in Kavango east region, Namibia. The final year nursing group of 2020 academic year had a population of 70 registered students at the time the study was conducted. The group was targeted because of its cultural diversity as evidenced by students from Namibian cultures such as Owambo, Rukwangali, Nyemba, Subiya, Gciriku and Nama as well as Shona from Zimbabwe.

Researchers used maximum variations purposive sampling technique to select participants of the study [18]. Maximum variation sampling involves purposefully selecting persons with variation on dimensions of interest. This was considered to allow for rich and diverse data needed to understand experiences of transcultural nursing at the intermediate hospital. Therefore, ensuring participants from diverse cultural backgrounds are represented in the sample. As advised by Polit and Beck [18], the researcher also use snowballing to ask early participants for referrals to students who are from unique and minority cultures in order to obtain different perspectives of transcultural experiences. In addition, other variations considered were sex and age groups as they are one of the factors determining cultural practices, beliefs, values, and norms [3].

The sample, as determined by data saturation consisted of 16 study participants. Of all participants approached to participate in the study, no one opted out or dropped out of the study. Researchers applied the following criteria in the sampling process:

1. Participants had to be available and agreed to participate in the study by signing informed consent form.
2. Nursing students from diverse cultural backgrounds.

To select participants, the second author approached final year students face to face during clinical practice and also by instant messages sent to their cohort-based WhatsApp group. On both approaches used to recruit participants, researchers explained the purpose and objectives of the study followed by signing written informed consents. To avoid conflicting with clinical practice schedules, students were given rights to choose date, time, and venue for face-to-face data collection. Options were also given for telephonic interviews as data were collected during the time Namibia recorded her first two COVID-19 positive cases in 2020.

### Data collection procedures

Data were collected in April to June 2020 through semi-structed individual interviews. A semi-structed interview guide was used to guide the data collection process. The interview guide was developed by the first and second authors, specifically for this study and is attached to this manuscript as supplementary file 1. Participants were asked to tell interviewers about their experiences of transcultural nursing during clinical practice, followed by prompts to understand their positive and negative experiences and how these impact on their experiences of clinical practice. The interview guide was piloted with two participants prior to the main data collection process. This assisted researchers on probing when responses were insufficient and not clear in order to seek for more clarity. Piloting was necessary to ensure high research quality when a depth of understanding is sought [19]. Participants who participated in pretesting the interview guide were not part of the main study, data obtained from the two interviews were analysed and form part of the main study.

The main interviews were conducted by the first and second authors, each with one participant at a time. No other people were present in the interview venue or listening to the conversations during telephonic interviews. Participants interviewed telephonically were phoned at a time agreed with them to minimize distractions and increase privacy [20]. All were conducted in English, and were audio recorded. In addition, field notes were made during and after the interviews to note body languages, non-verbal communication, researchers’ reflection and bracketing of own experiences. The duration of the interviews was determined by participants’ responses, lasted 38–55 min. Each interview was concluded when no new information emerged, and the interviewer included all prompts in the interview guide. All audio recording from the interviews were transcribed verbatim and were returned to participants for member checking before data analysis commenced.

### Data analysis

The second author transcribed the audio-taped interviews which were done verbatim. Tesch [21] eight steps in the coding process of qualitative data analysis guided the process which was manually done by the two authors. In step one, the second author read through all transcriptions carefully to understand the content, this was followed by step two, which involved writing down underlying meanings on the page margins. In step three, meanings on margins were given topic names and similar topics were clustered together into columns. This was followed by step four, writing down codes, then arranging them into categories. In step five, the author gave descriptive words to the topics and assembled all similar data materials that belong to each category together. In step six, names of topic were transformed into themes and sub-themes were made. In addition, field notes were read together with transcribed data. In step seven, themes and sub-themes were further refined, these represent preliminary findings of the study. In step eight, the first author repeated the same process of coding, then the two authors came together to agree on final themes. During data analysis, authors designed a coding tree for understanding and presentation of themes and how sub-themes emerged as well as codes that formed them. The third author validated the findings. Member checking was done by taking back transcriptions and themes to study participants, while audit inquiry was done as discussed in data quality criteria section of this article.

### Ethical considerations

The Declaration of Helsinki’s ethical principles for medical research involving human subjects were used as ethical guidance for this study [22]. In addition, participants gave their informed consent through signing a form preceding their participation in the study. As advised by Polit and Beck [18], participants were told that there would be no coercion if they wanted to opt out of the study, and that participation was voluntary. Participants could opt out of the study at any point and did not have to answer any questions they were not comfortable with. Inclusion criteria were met to ensure fairness. Numbers were allocated to participants instead of using their names, and no names were used in research findings, this ensured anonymity. Confidentiality was ensured by storing audio recordings on password-protected devices and only the researchers had access to them. Moreover, no other people listen to interview conversations during data collection [18]. The study was granted ethical clearance and permission by the departmental research ethics committee in the public university (ref 13/2019). In addition, ethical clearance was granted by the research unit in the Ministry of Health and Social Services (letter dated 06 January 2020).

## Findings

Ten female and six male nursing students participated, their age ranging from 22 to 31 years. Nyemba, Rukwangali and Aawambo cultural groups had three participants each, Subiya and Shona had two participants each, Gciriku, Mbukushu and Nama cultural groups had one participant each. Of the 16 participants, 14 were Namibian, while two were international students. Three participants were having prior nursing experience with a certificate in Nursing and midwifery, while thirteen participants were generic students, only with secondary education qualification. This difference is expected, in the study setting, the Bachelor of Nursing Science (clinical) honours programme admits a very limited number of candidates who apply through mature entry and recognition of prior learning. Four main themes and 12 subthemes emerged that reflected the experiences of nursing students on transcultural nursing during clinical practice and are presented in Table 1.

**Table 1:** Summary of study findings

Themes	Sub-themes
1. Nursing students’ exposure of different cultural practices and beliefs through transcultural nursing.	1.1. Communication practices during transcultural nursing 1.2. Role of gender and age in caring for a sick person experienced during transcultural nursing 1.3. Health care practices and beliefs related to western and traditional care systems.
2. Personal feelings experienced by nursing students during transcultural nursing	2.1. Positive feelings during transcultural nursing 2.2. Negative feelings during transcultural nursing.
3. Challenges experienced by nursing students during transcultural nursing	3.1. Language barriers during transcultural nursing 3.2. Lack of awareness on cultural practices, values and norms. 3.3. Cultural expectation of patients and resistance to health care interventions
4. Nursing students’ coping mechanisms during transcultural nursing	4.1. Being culturally competent 4.2. Peers support during transcultural nursing 4.3. Role of patients’ Charters of Rights during transcultural nursing

Source: Authors

### Theme 1: nursing students’ exposure of different cultural practices and beliefs through transcultural nursing

This theme describes the participants’ experiences of their exposure to cultural practices and beliefs of different clients and patients while they are in clinical settings. This includes communication practices, role of gender and age in caring for a sick person, and Western and traditional health systems’ practices and believes related to health care.

#### Communication practices during transcultural nursing

Participants indicated that in some cultures, the elders are the ones who greet young ones first while in other cultures the young ones greet the elders first, in both situations, it serves as a sign of respect. As far as greeting practices are concerned, in some cultures, hand shaking is preferred, while in some cultures, people prefer hugging each other, hand clapping and distance greeting. This implies that there are differences in greeting practices across different cultures. Moreover, nursing students also observed that some of their clients and patients prefer greeting in their own languages. This was mentioned by participants;

*I noticed some elderly patients gets annoyed when I greet them, then I realise ooh, I should wait for them to greet me (laughing). It’s like they expected me to come stand in front of them, they greet, then I greet back. (Participant 6)*

*There are so many differences especially manner of greeting, in my culture, wait for an elder to greet me, now here it’s like I am expected to greet first. (Participant 14)*

Participants revealed that at times, they are not sure as to whether keep eye contact with the patients/clients or not as it is perceived differently in different cultures depending on values and beliefs. This is because in some cultures, eye contact is emphasized, while it may be perceived as a sign of disrespect in some. This was mentioned;

*……. consulting an old patient and you are starring at her like straight into her eyes was like……. (shaking hands and smiling). You know in some cultures eye contact is a taboo, they might think you are disrespectful. (Participant 1)*

*In my culture, young people are prohibited from direct eye to eye contact with older people. Here at the hospital, one day an elderly man told me that I am not honest and I was probably under the influence of some drugs, just because I couldn’t keep eye contact, but you know its not allowed in my culture. (Participant 16)*

#### Role of gender and age in caring for a sick person experienced during transcultural nursing

Participants indicated that most patients are not comfortable to be examined by young nursing students, especially when it comes to examining private parts like genitals. Seeing nakedness of an elder person was associated with lack of respect in some cultures, while it is accepted in some cultures. This caused confusion as first year students are expected to perform basic nursing procedures, which include full wash of patients and providing a bed pan and urinals to patients not immobile patients. Moreover, participants have experienced that in some cultures, the young person is perceived as a novice and lack experience in whatever task they have to perform therefore, patients prefer to be cared for by mature nurses. Participants have experienced that some patients do not agree to be examined by nurses of the opposite sex, irrespective to whether the nurse is a student or a qualified cadre. The following was said by participants:

*‘…….but the old “tates” are always refusing to be injected by young female student nurses, but people from my culture were not a problem at all (seemingly worried). (Participant 10)*

*I remember in the last clinical rotation I was allocated in female ward, there was a woman who was bedridden but always refuse to be assisted by male nurses whenever she wants to change her clothes, dressing her wounds or bath. (Participant 12)*

*Eeeh one time when I was allocated in female ward, I was delegated to examine a certain patient who was almost 60 years old. The patient was a female so I had to examine the private parts because she was complaining of itching and she was saying she feels like she has developed some rash so the senior nurse ordered me to check the rashes. So, the patient refused because she felt like I am disrespecting her since um very young, I cannot just look or check her private parts just like that so yaa that’s the other challenge. And then another one,……. (long silent), that one was a male who refused to be examined by a female student nurse and that male was, he was young he was in his 20s so he refused to be examined by a female student nurse, yaa so that is something that I experienced. (Participant 6)*

#### Healthcare practices and beliefs related to western and traditional care systems

Participants revealed that there are different traditional herbs used by pregnant women and females in general for various reason. For example, pregnant women in some cultures take traditional herbs to speed up the labour process before coming to the hospital and some take herbs as they believe they minimizes labour pains. In some cultures, females also engage in vaginal packing whereby they put traditional herbs in the vagina to tighten it. Traditional herbs are also used in some cultures as means of family planning. The following was said by participants:

*I saw one pregnant women who arrive in labour room with an underwear full of greenish stuff, I enquired during admission and she told me her grandmother inserted them to like make the process quicker and also with just a little pain, I was like how……… and then……. She didn’t want to discuss further. (Participant 10)*

*One day I was working in family planning room whereby we asked all the females to show their pads in order to confirm whether they are on their periods those who want to start family planning whereby I came across with a lot of clients who did vaginal packing so you tell the patient like can I see your pad so you see the blood at least which is a sign of menstruation and the person will tell you ‘no I have a tampon’ so you ask the patient may you please remove the tampon for me at least to see the blood before you give the injection just to make sure the person is on her periods. Then the person will start telling you the truth that no there in a tampon I did put in herbs in my vagina and she will continue telling you that we do it traditionally to tighten the vagina traditionally so which is a new thing to me (participant 4).*

Use of traditional medicines was also discussed by nursing students as part of the experiences of transcultural nursing during clinical practice. Some patients use traditional medicines to supplement treatment they get from the hospital, some prefer to take traditional medicines instead of Westen medicines prescribed by medical doctors at health care facilities. There is a believe that traditional medicines are more effective for chronic illnesses such as epilepsy and some mental disorders.

*People like to use roots of trees, crushed leaves, dry herbs and dry animal skin as medicine to cure chronic illnesses, like that disease which make people to fall, and specific mental issues. They believe medicines from the hospital are slow acting and cause a lot of side effects. (Participant 8)*

Other practices are that some family members or even other patients prefer coming to different wards and pray for their relatives who are be admitted. Participants have observed while in clinical practice that some family members perform rituals on the patient’s bedside. This can be done to cure an illness or to prevent illness from spreading further. The following was said by participants:

***……….*** *during visiting time, family member or even patients from other wards may come and pray for the patients which is also happening in my place of origin.*

*One day I was allocated in paediatric ward, I noticed an elderly woman who can there with a small calabash with some powder, she started applying on the baby’s abdomen, the baby who was a patient. Ooh, I asked her what she was doing, she responded that it was their cultural rituals I must not interfere at all. I went back to the nurses’ post and didn’t tell anyone as I was afraid of the women, she looked like a “Sangoma” (looking warried) (Participant 5)*.

### Theme 2: personal feelings experienced by nursing students during transcultural nursing

Participants explained that as they were caring for patients from diverse cultures, they experienced different feelings that resulted from exposure to patients and clients’ different culture than oneself. Some experienced positive feelings while others experienced negative feelings.

#### Positive feelings during transcultural nursing

Some of the participants experienced positive feelings as they care for patients from diverse cultural backgrounds. These include feeling of confident, competent, proud, respected, and comfortable. This could be because, for some students it is first time coming closer to a person from a specific culture, therefore it arouses different feelings. Participants experienced feeling of being appreciated and accepted by the patients from different cultures, whom they rendered care to. Participants also felt welcomed especially by elderly patients who would show interest in talking to them. Some of the positive feelings experienced by participants are described below:

*It makes me feel more welcomed in Kavango region because when it comes to old people I mean old patients they show interest in us that we came to Kavango to help them so which is really nice. (Participant 3)*

*………. it makes you to be confident like you will be confident of meeting capable of you speaking with people from different background and you will be competent to react with them because of how you are used to working with different people that are coming from different cultural backgrounds. (Participant 8)*

#### Negative feelings during transcultural nursing

Participants experienced negative feelings as they cared for patients from different cultures. These include feeling unwanted especially by young female patients who claimed that students from other regions were taking their education and their jobs. Other negative feelings include nervousness, rejected, annoyed, sad, stressed, belittled, uncomfortable, incompetent, misunderstood, not trusted, and insulted. The following was mentioned during the interviews:

…….*at some point it made me feel belittled. It made me feel like maybe I am not competent enough or what. Even though I knew in my heart that I am competent since I can do this and that, I mean all procedures required at my level, I am able to do them (smiling). But because the patients were not comfortable with me treating them or with me eeh attending to them, it made me uncomfortable (participant 1).*

*Sometimes I feel nervous because you will always be too careful of whatever you are doing, you don’t know what’s right and wrong about their cultures, so you work with worries. (Participant 4)*

### Theme 3: challenges experienced by nursing students during transcultural nursing

Participants discussed challenges they face in caring for patients from different cultures whole in clinical practice. Some of these challenges interfere with learning processes in clinical settings, some served as lesson for future practices. Challenges revealed by students were related to language barriers, cultural practices, values, norms, and cultural expectations.

#### Language barriers during transcultural nursing

Language barrier was mentioned as a very big challenge to most participants because patients from different cultures speak their languages and therefore not able to communicate to nursing students from other cultural groups. This hinders nursing students from providing proper nursing care due to the reasons that they are not able to comprehend patients ’complains, progress and other subjective data required for health intervention. It was also revealed that nursing students came across patients who do not speak English, which they mostly use as an alternative language when dealing with a client or patient who speak different vernacular language. The feedback on services provided by nursing students was also described to be compromised due to language barrier. The following was described by the participants:

*The most difficult experience is language barrier. And because of language barrier most of the time I don’t know whether the service I am rendering is satisfactory. Sometimes it’s just difficult to talk to the patients, let’s talk about the primary health care clinics, when taking history and screening this someone and this someone is telling me about their complains, most of the time I don’t know whether um understanding these complains correctly. (Participant 3)*

*The worse part is that, I cannot get feedback on the service I provide to the clients because it’s not possible to speak to them. They cannot speak English and I cannot speak their languages and they cannot speak my language. (Participant 5)*

#### Lack of awareness on cultural practices, values and norms

Lack of awareness on cultural practices, values and norms was experienced as one of the barriers during transcultural nursing in clinical practice. For example, in some cultures, a patient is expected to clean his or her mouth with hot water before talking to anyone in the morning. Also, the openness about illness, personal belongings to keep on bedsides, ambulation of a sick person and mealtimes were challenges to nursing students because patients prefer different practices other than what is suggested by hospital routines. This was mentioned:

*In some cultures, they prefer a family member to do first baby bath and take care of the baby on the first day as this has serious consequences on the baby’s life if it’s done by a stranger. I was not aware (shaking hands). (Participant 16)*

*I once nursed a client who was refusing to remove her traditional necklace, its like she totally refused because according to her culture, it was so important for her to wear it at all times. But I didn’t know because in my culture we do not wear such items, I was just telling her to remove because it’s a policy when going for surgery. (Participant 9)*

#### Cultural-related expectation of patients and resistance to health care interventions

Participants indicated that patients from different cultures had different expectations towards their health as well as towards the care givers. For example, patients and clients expected nurses to be fluent in all languages spoken in the geographical region where they work. Some patients also expect to be admitted only a few days and go home to be taken care by relatives, while others expect to be admitted for long.

*One day I was told by a patient, how come you can’t speak Rukwangali when you are working in Kavango region. What kind of nurse are you? I was like no I am from another regions. (Participant 3)*

*A 36 years old woman discharged from maternity ward stayed for extra day because she thought it was too early to go home. Apparently in her culture, the man should not see her too soon after deliver, so she wanted to stay a bit longer. (Participant 14)*

Participant identified patients and clients’ resistance to health care interventions as part of their experience on transcultural nursing. For example, patients from some cultures have tendency to refuse medication, major operation, and some invasive procedures such as colonoscopy and haemorrhoidectomy and genital examination, regardless of explanation given. While for some, they accept interventions as long as proper explanation is given to them. This was mentioned during interviews:

*We used to have problems when it comes to giving medications to patients. They used to spit it out or to refuse just to refuse that I don’t want to take the medication. I can relate this to cultures because it was more common in some cultural groups. (Participant 2)*

### Theme 4: nursing students’ coping mechanisms during transcultural nursing

Participants described that as they are faced with different challenges in caring for patients from diverse cultural backgrounds, they had different ways which helped them to cope during transcultural nursing. Coping mechanisms of nursing students during transcultural nursing involve being culturally competent, getting support from peers, and use patients’ Charters of Rights as a guiding document.

#### Being culturally competent

Participants explained that learning different languages and a few cultural practises, norms and values related to health was very important as it facilitate communication and awareness of cultures, which may result in improved nurse-patient relation. It helped them to know patient’s beliefs and how they perceive things in order to understand different human behaviours associated with cultures. The following was described by some of the participants:

*………….to learn languages of different people from different cultural background and you will tend to learn some of their traditional things like what they value and what is considered a taboo. (Participant 3)*

Participants further indicated that they learn to be careful about what to say in front of patients and avoiding sensitive questions as well as being accommodative. Moreover, accepting cultural differences about health care and daily living was also mentioned as a coping mechanism during cultural nursing. Participants mention this:

…….*working with people from different cultures really has taught me to look at things differently not only in the way you consider them to be but in the you take things and see them in other perspectives things that you consider wrong maybe considered right or good in the other tribe or so. (participant 16)*

*…….also try to understand a patient better by understanding their background and where they come from. (Participant 14)*

#### Peers support during transcultural nursing

Participants revealed that providing care to patients from different cultures was made easy by support of peers. For example, by teaching each other different languages, teaching each other about different cultural practices, use other students as interpreters when a nursing student and patient speak different languages, in case a patient is not comfortable to be examined by students from opposite sex, the student of same sex would take over the task.

*In my clinical allocation group, I can say we are all from different cultural group, what we do we teach other basic terminologies during tea and lunch break. Yaa, I remember other day we discussed myths and taboos, cultural rituals and many others happening in Kavango region. (Participant 3)*

*I really help my male colleagues a lot, like with full wash of female bedridden elderly patients who refused to be seen there. (Participant 12)*

#### Role of patients’ charters of rights during transcultural nursing

Respect and dignity of patients was viewed as important aspects in the coping mechanisms during transcultural nursing. Participants expressed that allowing patients choices and autonomy in decision related to their health was one of the mechanisms utilized in transcultural nursing as well as adhering to privacy and confidentiality of patients, both in patients and outpatients. Allowing patients to be an active participant in promoting their own health, curative activities, prevention of further injuries or diseases and rehabilitation while admitted was one the aspects nursing students said to have experience during transcultural nursing. The following was explained by participants:

*When the patient says we don’t do this and that, just talk to them softly by explaining benefits so you obtain informed consent. (Participant 10)*

*We need to work together, the nurse and the patient must work together with the same goal helping the patient. Ask them to do something for themselves like exercising, going to other therapies other than just taking medicines. (Participant 1)*

## Discussion

In this study, students’ experiences on transcultural nursing during clinical practice were explored and described. It was evident that students’ experiences of transcultural nursing during clinical practice leads them to be exposed to different cultural practices and beliefs. Nursing students experienced personal feelings, challenges and have developed some coping mechanisms during transcultural nursing.

Due to patients and clients from diverse cultural groups nursing students coming in contact through clinical practice, participants in this study revealed that they are exposed to different cultural practices and beliefs. This may be seen as an advantage to nurses providing transcultural nursing because according to Paric et al., [23], being exposed to diversity is crucial to developing cultural competence. The findings on exposure to different cultures were also reported by Halabi and de Beer [24] who documented that students deal with patients from different cultures during their clinical training.

The use of traditional herbal medicine is common practice among urban and rural context in African continent. As far as traditional health practices are concerned, there are some childhood illnesses that are considered as not for Western healthcare services offered in hospitals, therefore requiring traditional and spiritual interventions [25]. This is similar in a current study where participants revealed that through transcultural nursing, they have seen patients using traditional medicines and perform rituals that are believed to be solutions to health issues they are experiencing or may be used to supplement medicines given at the hospital. Moreover, other traditional herbal medicines and cultural practices experienced by nursing students through transcultural nursing relates to care of pregnant women, post-partum period and care of the newborns. These findings were not surprising as multiple studies over the last decade reveal that pregnant women may take a variety of herbal medicines in forms of herbal extracts, crude herbal preparations, medicinal products of herbal origin, and dietary supplements consisting of proprietary blends of herbal medicines, minerals, and vitamins [26].

Another experience of students on transcultural nursing revealed in this study is the role of gender and age in caring for a sick person. In general, gender socialization and gender stereotypes may have influence on health-related behavior [27]. Asuquo and Akpan– Idiok [28] documented that caregiving is a primary role of female family members, which is designated by ideologies and is endorsed by cultures. In most situations, care is provided by adult female members, who are perceived as mature members of the family. This ideology could be causing patients to refuse care from young nursing students as they perceive them to be immature and inexperienced to care for a sick person, specifically if student and patient are of opposite sex. Similar findings were reported by Lilja and Tornerhjelm [29] who reported that due cultural beliefs, female participants were not allowed to bath a male patient. The student ends up making an alternative for another colleague to provide needed care. Although young adults aged 18 to 39 years reported to experience less physical strain and burden during care as compared to middle and older adults [30], the current study revealed that patients do not prefer to be cared for by young adults due to their cultural beliefs. Experience of different communication practices related to culture revealed in the current study was not surprising. Shirazi et al., [31] reported various interpersonal interaction across cultures, like lack of greeting, lack of eye contact, lack of physical touch and physical movement.

The personal experiences of nursing students during transcultural nursing include both positive and negative feelings. Positive feelings include confident, competent, proud, respected, welcomed, comfortable, appreciated and accepted by the patients from different cultures, whom they rendered care to. Similar findings were reported by Adamson [5] who documented feeling of encouragement, happy, wonderful, competent, appreciated and sense of belonging in students when nursing patients from other cultures. Notable was the negative feeling experienced by nursing students displayed through feeling unwanted especially by young female patients who claimed that students from other regions were taking their education and their jobs, nervousness, rejected, annoyed, sad, stressed, belittled, uncomfortable, incompetent, misunderstood, not trusted and insulted. These findings may be indicative of anxiety and fear due to the possibility of making mistakes and not correctly interpreting feelings, thoughts and wishes of the patients who are from different cultures [32].

It was evident in this study that students experienced some challenges during transcultural nursing. These challenges were language barriers, lack of awareness on cultural practices, values, and norms, as well as cultural expectations of patients and resistance to health care interventions. The language barrier experienced by students did not come as a surprise since Namibia is a multicultural society with more than 20 languages spoken by indigenous people. In addition, there are a lot of tourist attractions and business activities in town where study was conducted, this brings in people from other countries, who also receive healthcare services at the facilities where students do clinical practice. Previous research on experiences of newly qualified registered nurses and midwives in a rural health district in Namibia revealed cultural shock, language barriers and labelled as outsiders [33]. Therefore, this indicates diverse cultural groups pose challenges not only to nursing students but also to qualified nurses. Disrupted communication due to students and patients speaking different languages was also previously reported [29]. Although not revealed in the current study, language barriers lead to problems such as misinterpretations of information during counseling and health education sessions, delayed treatment, and medication errors [34].

According to Nur’ainun and Novieastari [35], the diverse places of origin of students do not hinder them in applying proper transcultural nursing. However, in the current study, the findings revealed that being from a diverse culture and place of origin was a challenge to provide care due to lack of awareness on cultural practices, norms, values, and beliefs. Similarly, Lilja and Tornerhjelm [29] also documented limited levels of knowledge of the different cultures in nursing students. These were major hindrances for nursing students providing care to diverse patients due to the reason that culture determines health care practices, beliefs and conceptions of health and illness. Cultural beliefs, norms, practices, and beliefs also determine the expectations of patients when they come to seek health care and therefore, determine their acceptability of health care interventions.

Individuals overcome stressful and uncomfortable situations by applying appropriate coping strategies. In transcultural nursing, nurses emphasize the importance of diverse language skills in order to be linguistically capable of understanding patients’ health issues and also to communicate with colleagues [34]. Although not revealed from the current findings, Larsen et al., [34] encouraged nurses to be prepared in the globalized health care system through nursing education. This is to adequately prepare nurse graduates to cope during transcultural nursing. Also, findings of the current study showed that nursing students cope by being culturally competent. They do it by being aware of their own culture, being aware there are different cultures in the context where they do clinical practice, improve their knowledge on different cultures, being sensitive to other cultures and have encounters with individuals from different cultures. The more nurses experience provision of care to patients from different cultures, the more they become culturally competent [36]. Peer support was also mentioned as a coping mechanism in the current study. This is because nursing students from schools located in multicultural societies tend to have more diverse students, like the case in the context of this study. This is an advantage since students may help each other with language translation, interpretations, and teaching on cultural practices. These help other students to understand patients’ needs and in return help to be culturally competent.

It was identified in the current study that Patients Charter of Rights has role to play in the coping mechanisms for nursing students during transcultural nursing. As an independent republic, Namibia has a documented patient charter policy which explains core values of the Ministry of Health and Social Services. It also consists of principles guiding public health and social care services, expectations for clients from health care providers and responsibilities of clients visiting health care facilities [37]. Therefore, to cope during transcultural nursing, students use the charter document to ensure the care provided does not violate basic human rights and is according to the guiding principles, which are applicable to all patients irrespective of their cultures.

The findings of the current study have significance in the local, national, and international contexts. For local and national context, Namibia is a multicultural society with 13 ethnic groups, which practice diverse cultures. In addition, due to migration, business activities and tourism, health care facilities have become multicultural entities. Thus, findings may assist training institutions to understand students’ experiences and help nursing students to cope during transcultural nursing since they are exposed to patients from diverse cultures. At international contexts, findings add to the body of knowledge on experiences of students during transcultural nursing, this may be helpful in enriching transcultural nursing courses in the curricula to help students while in clinical practice.

### Strengths and limitations of the study

The strength of the study lies in the fact that participants are from diverse cultural groups, which also include international students. Due to COVID-19 pandemic, this study utilized both telephonic and face to face interviews during data collection. Telephonic interviews were limited in observing facial expressions and other nonverbal communication cues that may display emotions such as fear, anger, sad and anxiety in participants and thus, limited researchers from identifying participants who might need emotional support, so this was considered as a study limitation.

### Recommendations

Recommendations are made for nurse education, clinical practice, and future research. For nursing education, students should be encouraged to write reflective essays on their exposure of transcultural nursing at the end of clinical practice rotation as this may help improve their future practice. Another recommendation for nursing education is that nursing schools located in multicultural societies should revise students’ selection criteria to ensure proportional representation of ethnic groups as well as international applicants. For clinical practice, the researchers recommended clinical supervisors to incorporate transcultural nursing issues into their routine meetings in the units as well as during students’ feedback time. Additionally, its recommended for future researchers to explore experiences of patients on care provided by nurses from diverse cultural backgrounds as well as experiences of qualified nurses on transcultural nursing to provide broader understanding on the topic.

## Conclusion

Due to globalization of healthcare, nurses and midwives throughout the world are increasingly expected to have encounters with patients, families and colleagues from diverse cultures and backgrounds, consequently, providing transcultural nursing. Through clinical nursing education practice, students become part of the healthcare and thus exposed to transcultural nursing. It was identified that students are exposed to different cultural practices and beliefs during transcultural nursing and also experience positive and negative personal feelings. They also experience some challenges related to language barriers, lack of awareness on cultural practices, norms, values and beliefs, cultural expectations of patients and resistance to health care interventions. As a result, students developed mechanisms to help them cope during transcultural nursing. These findings confirm the need to strengthen nursing education, clinical practice, and future research on transcultural nursing. The study has practical implications for nurses in clinical settings as it may provide baseline information to help students in becoming competent in providing transcultural nursing care. For nursing educators and professors, they may include students’ coping mechanisms during transcultural nursing in formal curriculum, and extra-curricular activities. Future researchers may consider quantitative assessment of nursing students’ cultural competence while in clinical practice.

### Electronic supplementary material

Below is the link to the electronic supplementary material.

Supplementary Material 1

## Data Availability

The data analysed during the study will be made available by the corresponding author upon reasonable request.

## Abbreviations

COVID-19: Corona virus disease 2019
