# Supplementary material for: Transcultural nursing: a qualitative analysis of nursing students’ experiences in a multicultural context in North-Eastern Namibia
Source: BMC Nurs. 2024 Feb 15;23:123. doi: 10.1186/s12912-024-01773-8 (PMC10870613; doi:10.1186/s12912-024-01773-8)
Supplement: Supplementary file 1 — Supplementary Material 1 [file 12912_2024_1773_MOESM1_ESM.docx]

**Supplementary file 1**

**Interview guide**

*Central question*

Tell me about your experience of transcultural nursing during clinical practice

*Prompts*

Tell me about your positive experience of transcultural nursing in clinical practice

Tell me about your negative experience about transcultural nursing in clinical practice

What was difficult or challenging? Why?

How did that impact on clinical practice?

How did that made you feel?

Tell me more about that
